# Viral Etiology of Influenza-Like Illnesses in Antananarivo, Madagascar, July 2008 to June 2009

**DOI:** 10.1371/journal.pone.0017579

**Published:** 2011-03-03

**Authors:** Norosoa Harline Razanajatovo, Vincent Richard, Jonathan Hoffmann, Jean-Marc Reynes, Girard Marcellin Razafitrimo, Rindra Vatosoa Randremanana, Jean-Michel Heraud

**Affiliations:** 1 Virology Unit, National Influenza Centre, Institut Pasteur de Madagascar (IPM), Antananarivo, Madagascar; 2 Epidemiology Unit, IPM, Antananarivo, Madagascar; University of Hong Kong, Hong Kong

## Abstract

**Background:**

In Madagascar, despite an influenza surveillance established since 1978, little is known about the etiology and prevalence of viruses other than influenza causing influenza-like illnesses (ILIs).

**Methodology/Principal Findings:**

From July 2008 to June 2009, we collected respiratory specimens from patients who presented ILIs symptoms in public and private clinics in Antananarivo (the capital city of Madagascar). ILIs were defined as body temperature ≥38°C and cough and at least two of the following symptoms: sore throat, rhinorrhea, headache and muscular pain, for a maximum duration of 3 days. We screened these specimens using five multiplex real time Reverse Transcription and/or Polymerase Chain Reaction assays for detection of 14 respiratory viruses. We detected respiratory viruses in 235/313 (75.1%) samples. Overall influenza virus A (27.3%) was the most common virus followed by rhinovirus (24.8%), RSV (21.2%), adenovirus (6.1%), coronavirus OC43 (6.1%), influenza virus B (3.9%), parainfluenza virus-3 (2.9%), and parainfluenza virus-1 (2.3%). Co-infections occurred in 29.4% (69/235) of infected patients and rhinovirus was the most detected virus (27.5%). Children under 5 years were more likely to have one or more detectable virus associated with their ILI. In this age group, compared to those ≥5 years, the risk of detecting more than one virus was higher (OR = 1.9), as was the risk of detecting of RSV (OR = 10.1) and adenovirus (OR = 4.7). While rhinovirus and adenovirus infections occurred year round, RSV, influenza virus A and coronavirus OC43 had defined period of circulation.

**Conclusions:**

In our study, we found that respiratory viruses play an important role in ILIs in the Malagasy community, particularly in children under 5 years old. These data provide a better understanding of the viral etiology of outpatients with ILI and describe for the first time importance of these viruses in different age group and their period of circulation.

## Introduction

Acute respiratory infections (ARI) are one of the leading causes of morbidity and mortality in infants and children, especially in developing countries. According to the World Health Organization (WHO), acute lower respiratory infections account for approximately 20% of all deaths among children under 5 years [1], and 70% of those deaths occur in Africa and Southeast Asia [2]. The elderly and the immune compromised persons are also at risk to develop serious complications due to ARI.

Several pathogens are implicated in ARI. Most of viral ARI especially in children are caused by human respiratory syncytial virus (RSV), which induces bronchiolitis, asthma exacerbation and pneumonia, and leads to high hospitalization rates [3], [4]. Influenza virus (FLUV) causes a common vaccine-preventable viral infection that can be severe or fatal in the very young, the elderly, and those with underlying illnesses. In temperate climates like in the U.S, wintertime seasonal influenza epidemics often result in dramatic increases in hospitalization, death and significant economic losses due to workplace absenteeism [5]. Human rhinovirus (HRV) is responsible for the majority of common colds causing upper ARI, and often induces pneumonia and asthma exacerbation in children [6], [7]. It was also observed that human metapneumovirus (HMPV) accounted for approximately 5–10% of all ARI in children and adults [8] and human adenovirus (HAdV) was shown to be responsible of 5–10% of ARI in children only [9]. In addition, infections caused by other viruses such as human parainfluenza viruses (HPIV), human coronaviruses HCoV-229E and HCoV-OC43 occur worldwide. Furthermore, the recently discovered human coronavirus HCoV-NL63 [10], HCoV-HKU1 [11] and human bocavirus (HBoV) [12] have also joined the panel of virus responsible of ARI.

Madagascar is an island located in the south-west part of the Indian Ocean. It has several bio-climates from sub-tropical to semi-arid along its coasts and a more temperate climate in the centre highlands. Madagascar is one of the poorest countries of the world with a health system that is hardly efficient to monitor and prevent outbreaks. Prospective influenza surveillance has been conducted in Madagascar through a network of sentinel sites for over 30 years. This surveillance system has enabled a better understanding of the epidemiology of influenza in the country [13]. Viruses isolated through surveillance have been used by WHO for its annual recommendation for influenza vaccines composition. Several studies about the burden of the Influenza virus in Madagascar were already published including the first investigation of outbreak of ILI which occurred in the central part of Madagascar in July 2002 [13], [14], [15]. However, little is known about the burden of the other respiratory viruses responsible for ILI among the Malagasy people. Even if some studies have demonstrated that ARI cause a high rate of hospitalization and mortality among children in Madagascar [16], [17], only one has reported data regarding respiratory viruses detected in children hospitalized with ARI syndromes [17]. In that study, viral infection were detected in 43/80 (54%) of children under 10 years old.

In order to fill this gap and to better understand the epidemiology of ILI in Madagascar, we investigated the etiology and the prevalence of selected viral respiratory pathogens in patients visiting our sentinel sites for ILI, by using multiplex real-time Reverse Transcription Polymerase Chain Reaction (rRT-PCR) assay for RNA viruses and real-time Polymerase Chain Reaction (rPCR) assay for DNA viruses. These assays allowed the simultaneous detection of a large panel of respiratory viruses.

## Materials and Methods

### Ethics Statement

The protocol and oral consent were determined as routine surveillance activity, and therefore non-research by the National Steering Committee for Surveillance of Fevers, an entity representing MoH, IPM, WHO and Clinicians in compliance with all applicable National regulations governing the protection of human subjects. Data were collected in an objective of surveillance and are anonymous. Collections of non-sensitive data or an observation from normal care in which participants remain anonymous do not require ethics committee review. Only physician could withdraw anonymity. Before each sample, physician gave information about surveillance system and objectives to prevent disease spread in community. After that, patients could refuse to participate and no specimen will be taken. Oral consent was documented in the patient form.

### Study design and settings

We conducted a study in 9 influenza sentinel sites in Antananarivo, the capital of Madagascar located in the central part of the country. Two distinct seasons exist in the central region: a cold and dry season (austral winter) from May to October and a warm and rainy season (austral summer) between November and April. Influenza sentinel sites were private and public primary health care centers and part of a National Surveillance System that monitored fever syndromes [18].

### Study subjects and specimens

We identified patients at consultation presenting with an ILI according to a modified WHO standard case definition [19]. We defined ILI as the presence of fever (body temperature ≥38°C) and cough and at least two of the following symptoms: sore throat, rhinorrhea, headache and muscular pain, for a maximum duration of 3 days. From each case, we collected demographic, clinical and epidemiological data as well as nasopharyngeal and/or oropharyngeal swabs. The respiratory specimens were placed in Universal Transport Media (Copan, Italia), and transported at 4°C twice a week from sentinel sites to the National Influenza Center. Specimens were stored at −80°C before processing. In this retrospective study, we analyzed anonymous specimens collected from July 2008 to June 2009.

### Detection of respiratory viruses (see supplementary File S1 for technical details)

For the study, viral RNA and DNA were extracted from specimens. We then performed four multiplex rRT-PCR and one multiplex rPCR assays using TaqMan technology targeting 14 respiratory viruses: HPIV-1, -2, -3 for multiplex 1; HCoV-OC43, HRV and FLUBV for multiplex 2; HMPV, RSV and FLUAV for multiplex 3; HCoV-229E, HCoV-HKU1, HCoV-NL63 for multiplex 4 and HAdV and HBoV for multiplex 5 (see supplementary Table S1 for sequences of primers and probes). Sample was considered as positive when its Ct value was equal or above the Ct value of the Limit of Detection (LOD) of the corresponding reference viruses. For characterization of influenza A subtypes, specimens were inoculated in MDCK cell line and then identified according to the WHO protocol (WHO/CDS/CSR/NCS/2002.5 Rev.1).

### Data analysis

We analyzed the demographic and clinical characteristics of the study subjects and the positive cases as well as the seasonal patterns of the most common respiratory viruses. We compared the clinical characteristics of patients infected by FLUAV, HRV and RSV to those of all virus positive patients. The Chi-square test and Fisher's exact test were used for univariate analysis, with the ANOVA and Kruskall Wallis tests used for comparison of medians. P-values<0.05 were considered to be statistically significant. Explanatory variables associated with a p-value less than 0.20 were analyzed by logistic regression to investigate the confounding factors. Only predictors significant at α = 0.05 were included in the final multivariable model. Analysis was performed using R version 2.7.0 software (R Development Core Team. R: A language and environment for statistical computing. R Foundation for Statistical Computing, 2009, Vienna, Austria. ISBN 3-900051-07-0, URL http://www.r-project.org.).

## Results

### Demographics and clinical characteristics of outpatients

From July 2008 to June 2009, we obtained 313 samples from patients suffering from ILI (Table 1). There were 138 (44.1%) males and 175 (55.9%) females (ratio M/F = 0.79). Among all patients tested, 235 (75.1%) were positive for at least one pathogen. Single infections occurred in 166 (53.0%) patients and co-infections were detected in 69 (22.0%) patients. No difference was observed between male and female regarding rate of infections.

**Table 1:** Demography and clinical characteristics of outpatients presenting ILI relating to infections in Antananarivo, from July 2008 to June 2009.

Characteristics	ILI (%)	Infected (%)		
	Total	Total	Single infection	Co-infection
	N = 313	N = 235	N = 166	N = 69
**Sex**				
Male	138 (44.1)	104 (44.3)	75 (45.2)	29 (42.0)
Female	175 (55.9)	131 (55.7)	91 (54.8)	40 (58.0)
**Age group (years)**				
0–4	154 (49.2)	127 (54.0)	82 (49.4)	45 (65.2)
5–9	41 (13.1)	30 (12.8)	23 (13.9)	7 (10.2)
10–14	25 (8.0)	16 (6.8)	12 (7.2)	4 (5.8)
15–19	17 (5.4)	9 (3.8)	5 (3.0)	4 (5.8)
> = 20	76 (24.3)	53 (22.6)	44 (26.5)	9 (13.0)
**Clinical symptoms** a				
Sore throat	294 (93.9)	220 (93,6)	155 (93,4)	65 (94,2)
Rhinorrhea	191 (61.0)	150 (63,8)	98 (59,0)	52 (75,4)
Headache	124 (39.6)	93 (39,6)	64 (38,6)	29 (42,0)
Muscular pain	55 (17.6)	79 (33,6)	67 (40,4)	12 (17,4)

N = total number of patient.

a all patient had fever and cough at presentation as inclusion criteria.

Age ranged between 3 months and 77 years (median: 10 years). Children <5 years old represented 49.2% of all ILI cases. Viral infections occurred in all age groups. Nevertheless, the highest percentage of viral infections was observed in children under 5 years old (54.0%). In this group single and co-infections represented 49.4% and 65.2% respectively. In univariate analysis, co-infection were statistically different (P-value = 0.02) in age group <5years (35.4%) than in age group ≥5years (22.2%) (OR = 1.9, 95%CI:[1.1–3.6]).

All patients presented with fever and cough as they are inclusion criteria. Sore throat was present in 93.9% of the patients, followed by rhinorrhea (61.0%), headache (39.6%), and muscular pain (17.6%). Analysis of clinical symptoms according with co-infection versus single infection adjusted with age group found no statistically difference between patients with single infection and patients with co-infection.

### Virus detection

Among all positive specimens, single infection occurred in 166 (70.6%) outpatients while co-infections were detected in 69 (29.4%) outpatients. FLUAV, HRV and RSV were the most common viruses detected with respectively 85 (27.3%), 77 (24.8%) and 66 (21.2%) positive specimens (Table 2). HAdV and HCoV-OC43 were detected in 19 samples (6.1%). Regarding other viruses, their representativeness ranged from 3.9% to 0.6%. No HPIV-2 was detected during our study. HRV, FLUAV and RSV were involved in the majority of co-infections with respectively 40 (27.7%), 35 (24.1%) and 24 (16.6%) of total viruses detected (145). Five (1.6%) triple infections and one quadruple infection (0.3%) were occurred.

**Table 2:** Distribution of viruses related to infections in Antananarivo, from July 2008 to June 2009.

Virus	Total (%)		Single infection (%)		Co-infection (%)	
	<5 yr	> = 5 yr	<5 yr	> = 5 yr	<5 yr	> = 5 yr
	N = 177	N = 134	N = 82	N = 84	N = 95	N = 50
FLUAV	40 (22.6)	45 (33.6)	21 (25.6)	29 (34.5)	19 (20.0)	16 (32.0)
HRV	38 (21.5)	39 (29.1)	12 (14.6)	25 (29.8)	26 (27.4)	14 (28.0)
RSV	54 (30.5)	12 (8.9)	35 (42.7)	7 (8.3)	19 (20.0)	5 (10.0)
HAdV	13 (7.3)	6 (4.5)	8 (9.8)	4 (4.8)	5 (5.3)	2 (4.0)
HCoV-OC43	7 (3.9)	12 (8.9)	1 (1.2)	6 (7.1)	6 (6.3)	6 (12.0)
FLUBV	5 (2.8)	7 (5.2)	1 (1.2)	6 (7.1)	4 (4.2)	1 (2.0)
HPIV-3	5 (2.8)	4 (3.0)	1 (1.2)	3 (3.6)	4 (4.2)	1 (2.0)
HPIV-1	5 (2.8)	2 (1.5)	0 (0.0)	1 (1.2)	5 (5.3)	1 (2.0)
HCoV-229E	3 (1.7)	2 (1.5)	1 (1.2)	0 (0.0)	2 (2.1)	2 (4.0)
HMPV	3 (1.7)	1 (0.7)	1 (1.2)	0 (0.0)	2 (2.1)	1 (2.0)
HCoV-NL63	1 (0.6)	3 (2.2)	0 (0.0)	2 (2.4)	1 (1.1)	1 (2.0)
HCoV-HKU1	1 (0.6)	1 (0.7)	0 (0.0)	1 (1.2)	1 (1.1)	0 (0.0)
HBoV	2 (1.1)	0 (0.0)	1 (1.2)	0 (0.0)	1 (1.1)	0 (0.0)
HPIV-2	0 (0.0)	0 (0.0)	0 (0.0)	0 (0.0)	0 (0.0)	0 (0.0)

N = total number of virus detected.

RSV was the most frequent virus detected in children less than 5 years with ILI symptoms (30.5%), followed by FLUAV (22.6%) and HRV (21.5%). Analyses of co-infection pairs showed that HRV with RSV or FLUAV are the most common paired viruses with respectively 15 and 14 cases followed by FLUA with RSV (8 cases) or HCoV-OC43 (5 cases) (Supplementary Table S2). We introduced variables indicating viruses in an initial logistic regressive model. Multivariate analysis showed viruses independently linked to age group less than 5 years old. Indeed, the probability was significantly higher for RSV (OR: 10.1, 95%CI: [4.4–24; 1]) and HAdV (OR: 4.7, 95%CI: [1.4–10.4]).

In the multivariate analysis adjusted with age group, including clinical symptoms (sore throat, headache, muscular pain and rhinorrhea), outpatients with rhinorrhea were more likely to have RSV infection (OR = 14.7, 95%CI:[5.4–40.3]), while it was significantly less present in outpatients infected with FLUAV (OR = 0.4, 95%CI:[0.2–0.7]). No clinical symptoms were specifically associated with HRV infection.

### Seasonality

**Figure 1:**
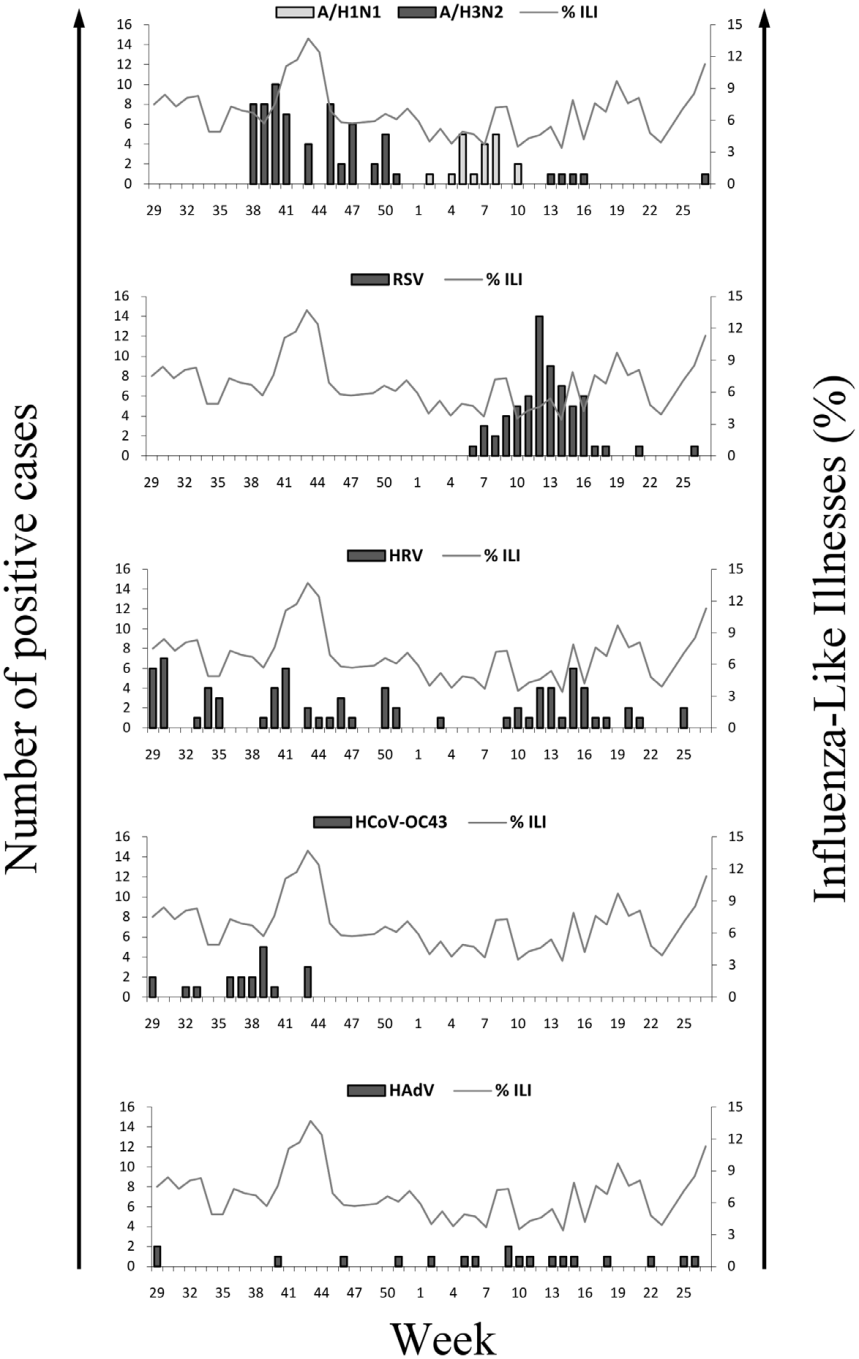
Incidence of the most frequent respiratory viruses detected in Antananarivo, from July 2008 to June 2009. Specimens from outpatient (n = 313) that consult for ILI were analyzed using multiplex rRT-PCR (FLUAV, RSV, HRV and HCoV-OC43) and rPCR (HAdV). Influenza A subtyping (A/H3N2 and A/H1N1) were determined after cell culture. Each panel shows the weekly incidence of one virus. For each virus, bars represent the number of specimen detected. The curve showing the weekly proportion of ILI among total visit based on data collected from one sentinel site during the study period was added to each panel.

During the study year, we observed virus circulation throughout the period. However, increased viral activity was observed in September–October 2008 and in March–April 2009. Figure 1 shows the seasonal patterns of the most prevalent viruses: FLUAV, HRV, RSV, HCoV-OC43 and HAdV. Seasonal FLUAV was detected during three distinct periods. The first period of circulation occurred from October to December 2008. This period was dominated by A/H3N2 subtype and coincided with an increase of ILI cases. The second wave of FLUAV occurred from January to March 2009 and it was dominated by A/H1N1 subtype. This period was followed by a low level circulation of A/H3N2 in April 2009. RSV was detected only from February to May with a peak in March. HRV and HAdV were detected throughout the study period. Higher HRV activity coincided with a decrease in FLUAV circulation, while HAdV was more frequent during the concomitant circulation of FLUAV and RSV from February to May. HCoV-OC43 was detected from July to October, and mostly before the FLUAV season. The other respiratory viruses appeared sporadically during the study period.

## Discussion

Our study demonstrated that respiratory viruses are often found in patients that presented to outpatient clinics with ILI in Madagascar. Respiratory viral pathogens were present in 75.1% of our samples, which is largely higher than other studies in which from 37% to 48% of the samples were positive [20], [21], [22], [23]. These differences could be due to seasonality, environmental factors, socio-economic status and certainly to the high specificity of our inclusion criteria. Indeed, in the study by Brittain-Long et al. all respiratory infections regardless of symptoms were included; Druce et al. included hospitalized cases in their study only for 5 months of the year. In our study, we defined ILI as fever and cough with at least two of listed symptoms (sore throat, rhinorrhea, headache and muscular pain, for a maximum duration of 3 days). Among infected patients, FLUAV (27.3%), HRV (24.8%) and RSV (21.2%) were the most common pathogens detected.

We found that patients presenting to clinics with ILIs were more frequently children less than 5 years (49.2%). Viral etiology was also more frequent in patient in this age group (54.0%) and co-infections were statistically more frequent when compared to patient ≥5 years (P-value = 0.02). These data were very similar to a previous study from Ravaoarinoro et al. in which 54% of ARI in hospitalized infants less than 10 years had a viral etiology [17]. We found that RSV was the most common virus in children under 5 years. The likelihood of detecting RSV was higher in this age group, compared to those ≥5 years (OR = 10.1). The importance of RSV as etiological agent for ILIs in children might have an impact in hospitalized children with upper and lower respiratory tract infections as observed by El Hajjee et al., and Dicarlo et al. [3],[4]. A recently published study reported that RSV associated with lower ARI accounted for 33.8 millions in 2005, which 96% occurred only in developing countries [24]. Our study did not address the burden of RSV infections within the Malagasy community; nevertheless, an ongoing study is currently screening SARI cases at two hospitals sentinel sites.

The observed influenza (FLUAV and FLUBV) prevalence in patient presenting with ILIs is similar to previous data from the National Influenza Center in Madagascar. Indeed, since 2006, rates of influenza positive cases among all specimens received ranged from 27.8% to 35.8% (unpublished data). HRV was also one of the most common pathogen in children under 5 years. HRV is known to be responsible for upper ARI but also in bronchiolitis and asthma exacerbation in infants and children [6], [7].

We detected few HMPV from patient with ILI. Three out of 4 were children under 5, all suffering from upper ARI. This low prevalence of HPMV infections in children is in agreement with previous study which found that HMPV is less prevalent in infants suffering from upper than lower ARI [25]. However, Pabbaraju et al. showed a high prevalence of HMPV in upper than in lower ARI [26]. Since we observed high prevalence of picornaviruses like HRV, it could lead to a decrease of the frequency of HMPV as suggested by Williams et al. [25].

Our findings that HCoV-OC43 and HAdV was present in 6.1% of the positive specimens suggest that these viruses could be important pathogens in ILIs within the community. In particular, we found that HAdV were more frequent among children under 5 years that present ILIs (OR = 4.7). However, further studies including a greater number of samples are needed to underline the burden of these two viruses in respiratory diseases in Madagascar.

Interestingly, we observed viral co-infections rates (29.4%) higher than reported in previous studies [20], [21]. The sensitivity of the diagnostic method and the large proportion of samples from children may play a role in the observed frequency of co-infections. Moreover, several viruses circulated with a similar seasonal pattern; those viruses tended to cause the most co-infections. In our study, the prevalence of viral infections (single and co-infection) were higher in children under 5 years (49.4% and 65.2%) as expected, since level of pre-immunity in this age group is lower.

We observed viral activity throughout our study period with two distinct peaks: one in winter, which corresponds with the cold and dry season, and one in late summer, which occurs during the rainy season. In Antananarivo, influenza circulation occurred during two distinct periods. The first one during the cold season and similar to what occurs in southern hemisphere countries. Considering that Madagascar is in the southern hemisphere, the second circulation from January to March is particular. Indeed, it is concomitant to influenza circulation in countries with temperate climate from the northern hemisphere. Our hypothesis is that this circulation could be linked to the increase of travelers between Europe (France) and Madagascar during Christmas holidays. Nevertheless, this pattern of influenza circulation is also observed in other subtropical regions like in Hong-Kong [13], [27], [28]. A circulation of RSV was occurred during the warm and rainy season from February to May with a peak on March. RSV infection occurred in Madagascar as observed in other countries with subtropical climate [6],[29],[30]. A study in the Nepal (Kathmandu Valley) showed a positive correlation between RSV and humidity [29]. Another one in Kenya (Kilifi District) detected two epidemics of RSV infection during rainy and warmer season showed no correlation between RSV circulation and meteorological factors [31]. Nevertheless, climate in Kilifi is quite different compare to one in Antananarivo. Further studies including meteorological parameters are needed to understand if temperature and/or humidity are factors associate with RSV infection in Madagascar. Indeed, differences might be observed when compare viral circulation in center highland (subtropical, temperate) to coastal area (equatorial to semi-arid). HRV was detected all year around as observed by Pierangeli et al. and Matthew et al. [6], [7]. In our study, HCoV-OC43 infection occurred in winter, before the FLUAV circulation, however HAdV was more frequent during summer. The other respiratory viruses showed a low prevalence during the study period, without any defined seasonal pattern.

Our study has some limitations. The limited sample size of this study did not allow the thorough investigation of association of symptoms in relation to infection with the detected pathogens. Moreover, by comparing the proportion of samples in each age group with the age pyramid of Madagascar, we found that our sample was biased toward young patients. Thus, even if it is known that children are more susceptible to respiratory infections we cannot assess if a particular age population is more at risk of getting a specific infection and co-infection. In addition, our sentinel surveillance in Madagascar was not population-based, so we could not extrapolate prevalence data from clinics to the general population. This study is based on influenza sentinel surveillance, thus our case definition for ILI is strict and excludes many viral infections with no fever or asymptomatic cases. Taken this into account, it is likely that with a broader definition, we would have detected more viral infection. On the other hand, since Real-Time PCR is very sensitive and can detect low viral load and does not required replication-competent viruses, we could not exclude that some detections could be due to carriage or co-carriage in the case of co-infections. Indeed some authors have shown that some respiratory viruses like HRV, HADV and HCoV can be detected in asymptomatic children [32] and that RNA from HRV can be detected from nasal mucus of children several weeks after the onset of the disease [33]. Thus, some viruses that we detected might not cause the disease. On the other hand we can also argue that, in some cases, it is the co-infection that causes the disease. In any case, previous studies showing good sensitivities and specificities for symptomatic patients [34], [35], suggest that molecular assays testing for these fourteen pathogens in respiratory secretions still have some interests in clinical practice.

The viruses temporal patterns detected in this study also need to be assessed over multiple years to identify long-term seasonal patterns. To end, this study did not test for bacteria, and therefore gaps remain in our understanding of all the etiologies of ILIs in Madagascar.

In conclusion, the results of our study give us a better understanding of the viral etiology of current ILI cases in Madagascar. This may lead to more appropriate care and treatment of patients including the correct application of antibiotics. Moreover, the use of multiplex rRT-PCR and rPCR permit a rapid differential diagnosis of ILI cases potentially enabling rapid detection and response to outbreak.

## Supporting Information

File S1 Laboratory Details.
(DOC)

Table S1 Sequences of Primers and Probes used in multiplex real-time RT-PCR and PCR assays.
(DOC)

Table S2 Distribution of age groups related to each type of major co-infections.
(DOC)
