# Supplementary material for: Viral Etiology of Influenza-Like Illnesses in Antananarivo, Madagascar, July 2008 to June 2009
Source: PLoS One. 2011 Mar 3;6(3):e17579. doi: 10.1371/journal.pone.0017579 (PMC3048401; doi:10.1371/journal.pone.0017579)
Supplement: File S1 — Laboratory Details. (DOC) [file pone.0017579.s001.doc]

**File S1: Laboratory Details**

Materials and Methods:

**Nucleic acid extraction**

For the study, viral RNA and DNA was extracted from 140 µl of each sample, using the QIAamp viral RNA mini kit and QIAamp MinElute virus kit (QIAgen, Courtaboeuf, France) according to manufacturer’s protocol. Whenever possible, we tested immediately after extraction. Otherwise, we stored the DNA/RNA at −80°C until analysis.

**Positive controls**

We obtained HPIV-1 (VR-94), HPIV-2 (VR-92), HPIV-3 (VR-93), HCoV-OC43 (VR-1558), RSV (VR-1580) and HAdV (VR-1572) from the American Type Culture Collection. Positive controls for HRV-A, -B and C was kindly provided by the Virology laboratory, University Hospitals of Geneva, Switzerland. HMPV, HCoV-229E, HCoV-HKU1, HCoV-NL63 were kindly given by the Department of Clinical Microbiology and Infection Control, Hospital of Halmstad, Sweden. HBoV control was provided by the Institute of Virology and Immunobiology, University of Wuerzburg, Germany. FLUAV and FLUBV were provided by our laboratory after confirmation by the WHO Collaborative Center in London (National Institute for Medical Research, NIMR).

**Primers and Probes**

We used primers and probes validated in other studies for amplifying FLUAV, RSV, HMPV, HPIV-1, -2, -3, HCoV-OC43, HCoV-229E, HCoV-HKU1, HCoV-NL63, HAdV, and HBoV. (FLUAV probe was provided by the National Reference Center for Influenza Viruses, Institut Pasteur, Paris). For HRV and FLUBV, we designed primers and probes after aligning respectively 50 and 20 sequences of representative strains of each pathogen from NCBI database using ClustalW (BioEdit Sequence Alignment Software Version7.0.9). For HRV, probe was designed to be specific for HRV strains. We test the specificity of our probe against enteroviruses (EVs) commonly isolated from the national reference laboratory for polioviruses. None of the EVs tested were detected using our probe. For influenza B we validated our primers and probes with both Victoria and Yamagata lineage strains that circulated during recent years in Madagascar. The sequences of primers and probes as well as each targeted gene are reported in Table S1.

**Multiplex real time RT-PCR and PCR (rRT-PCR, rPCR)**

We performed four multiplex rRT-PCR and one rPCR assays using TaqMan technology targeting 14 respiratory viruses: HPIV-1, -2, -3 for multiplex 1; HCoV-OC43, HRV and FLUBV for multiplex 2; HMPV, RSV and FLUAV for multiplex 3; HCoV-229E, HCoV-HKU1, HCoV-NL63 for multiplex 4 and HAdV and HBoV for multiplex 5. For the detection of RNA viruses, we performed rRT-PCR as a single step using the Superscript III Platinium One-step Quantitative RT-PCR System (Invitrogen, California, USA). Briefly, the reaction mixture contained 12.5 µL of 2× Reaction Mix, 0.2 µM of each primer and probe, 2 mM of MgSO4 and 0.6 µL of Superscript III RT Platinium TaqMix. For DNA viruses, we performed rPCR using TaqMan Universal PCR Master Mix (Applied Biosystem, New Jersey, USA). In that assay, the reaction mixture contained 12.5 µL of TaqMan 2X PCR Master Mix, 0.2 µM of each primer and probe. For all assays, a 2.5 µL of RNA or DNA extract and Nuclease-free water were added to give a final volume of 25 µL. The thermal cycling was implemented using Rotorgene 6000 (Corbett Life Sciences, Mortlake, Australia) with a reverse transcription step at 50°C for 30 min followed by a 2 min hold at 95°C for RNA viruses. For DNA viruses, thermal cycling started with a first hold at 50°C for 2 min followed by a 10 min hold at 95°C. Amplification consisted in 45 cycles of denaturation at 95°C for 15 seconds and annealing-extension at 55°C for 30 sec for RNA viruses or 60°C for 60 sec for DNA viruses. We amplified each sample in 5 reactions, each containing primers and probes for 2 or 3 different targets. For each target, limit of detection (LOD) was assessed using 10-fold dilution series of reference viruses (i.e. positive controls). The LOD corresponds to the highest dilution of the control that gave a positive result. Sample was considered as positive when its Ct value was equal or above the Ct value of the LOD of the corresponding viruses. LOD of reference viruses ranged from Ct=36 to Ct=38. Specific detection was observed for each virus by using different fluorescent probes within the same multiplex. We observed amplification only in the presence of RNA/DNA derived from the specific virus. No amplification occurred in the non template control as well as negative controls, which contained irrelevant RNA/DNA. We also did not observe any difference in Ct values of virus amplified alone versus multiplex assays and LODs demonstrated a similar sensitivity between the single virus and the multiplex assays.

**Influenza Viruses subtyping**

As part of our routine influenza surveillance, all specimens received at the National Influenza Center are inoculated in MDCK cell line for typing and subtyping. FLUAV were then identified according to the WHO protocol (WHO/CDS/CSR/NCS/2002.5 Rev.1) using the WHO Influenza Reagent Kit for identification of influenza isolates (CDC, Atlanta, GA, USA).
