# Supplementary material for: Viral Etiology of Influenza-Like Illnesses in Antananarivo, Madagascar, July 2008 to June 2009
Source: PLoS One. 2011 Mar 3;6(3):e17579. doi: 10.1371/journal.pone.0017579 (PMC3048401; doi:10.1371/journal.pone.0017579)
Supplement: Table S1 — Sequences of Primers and Probes used in multiplex real-time RT-PCR and PCR assays. (DOC) [file pone.0017579.s002.doc]

**Table S2:** Sequences of Primers and Probes used in multiplex real-time RT-PCR and PCR assays.

| **Multiplex** | **Virus Type** | **Target Gene (position)** | **GenBank Accession No.** | **Oligonucleotide sequence (5’ to 3’)^a^** | **Origin** |
| --- | --- | --- | --- | --- | --- |
| **1** | HPIV-1 | Hemagglutinin-Neuraminidase | U70948 | Fwd: GTTGTCAATGTCTTAATTCGTATCAATAATT | [30] |
|  |  | (1191-1299) |  | Rev: GTAGCCTMCCTTCGGCACCTAA |  |
|  |  |  |  | Pr: (6-FAM)-TAGGCCAAAGATTGTTGTCGAGACTATTCCAA-(TAMRA) |  |
|  | HPIV-2 | Hemagglutinin-Neuraminidase | D00865 | Fwd: GCATTTCCAATCTTCAGGACTATGA | [30] |
|  |  | (767-856) |  | Rev: ACCTCCTGGTATAGCAGTGACTGAAC |  |
|  |  |  |  | Pr: (JOE)-CCATTTACCTAAGTGATGGAATCAATCGCAAA-(BHQ-1) |  |
|  | HPIV-3 | Hemagglutinin-Neuraminidase) | EU814626 | Fwd: TGATGAAAGATCAGATTATGCATATC | [31] |
|  |  | (822-1070 |  | Rev: CCGCGACACCCAGTTGTG |  |
|  |  |  |  | Pr: (ROX)-TGGACCAGGGATATACTACAAAGGCAAAAT-(BHQ-2) |  |
| **2** | HCoV-OC43 | Nucleocapsid protein | AY391777 | Fwd: CGATGAGGCTATTCCGACTAGGT | [32] |
|  |  | (29607-29682) |  | Rev: CCTTCCTGAGCCTTCAATATAGTAACC |  |
|  |  |  |  | Pr: (ROX)-TCCGCCTGGCACGGTACTCCCT-(BHQ-2) |  |
|  | HRV | 5' Noncoding region | EU840952 | Fwd: AGTCCTCCGGCCCCTGAAT | This publication |
|  |  | (441-558) |  | Rev: ACACGGACACCCAAAGTAGT |  |
|  |  |  |  | Pr: (CY5)-TGAGCAATTGTGGGATGGGA-(BHQ-2) |  |
|  | FLUBV | Hemagglutinin | FJ152012 | Fwd: AAATACGGTGGATTAAATAAAAGCAA | This publication |
|  |  | (937-1106) |  | Rev: CCAGCAATAGCTCCGAAGAAA |  |
|  |  |  |  | Pr: (JOE)-CACCCATATTGGGCAATTTCCTATGGC-(BHQ-1) |  |
| **3** | HMPV | Nucleocapsid protein | AY145286 | Fwd: ATGTCTCTTCAAGGGATTCACCT | [20] |
|  |  | (1-139) |  | Rev: AMAGYGTTATTTCTTGTTGCAATGATGA |  |
|  |  |  |  | Pr: (JOE)-CATGCTATATTAAAAGAGTCTCARTAC-(BHQ-1) |  |
|  | RSV | Matrix protein | AY353550 | Fwd: GCAAATATGGAAACATACGTGAACA | [20] |
|  |  | (3255-3369) |  | Rev: GCACCCATATTGTWAGTGATGCA |  |
|  |  |  |  | Pr: (ROX)-CTTCACGAAGGCTCCACATACACAGCWG-(BHQ-2) |  |
|  | FLUAV | Matrix protein | DQ508860 | Fwd: CTTCTAACCGAGGTCGAAACG | [33] |
|  |  | (7-251) |  | Rev: AGGGCATTTTGGACAAAKCGTCTA |  |
|  |  |  |  | Pr: (6-FAM)-CCTCAAAGCCGAGATCGCGCA-(BHQ-1) |  |
| **4** | HCoV-229E | Nucleocapsid protein | AF304460 | Fwd: CAGTCAAATGGGCTGATGCA | [34] |
|  |  | (25712-25768) |  | Rev: AAAGGGCTATAAAGAGAATAAGGTATTCT |  |
|  |  |  |  | Pr: (6-FAM)CCCTGACGACCACGTTGTGGTTCA(TAMRA) |  |
|  | HCoV-HKU1 | Nucleocapsid protein | AY597011 | Fwd: ATCTGARCGAAAYYAYCAAAC | [35] |
|  |  | (28409-28847) |  | Rev: CGYAAACCTAGTAGGGATAGCTT |  |
|  |  |  |  | Pr: (CY5)CCCATTGCTTWCGGRATACCCCCTT(BHQ-2) |  |
|  | HCoV-NL63 | Nucleocapsid protein | AY563107 | Fwd: GACCAAAGCACTGAATAACATTTTCC | [34] |
|  |  | (360-469) |  | Rev: ACCTAATAAGCCTCTTTCTCAACCC |  |
|  |  |  |  | Pr: (JOE)AACACGATTCCAACGAGGTTTCTTCAACTGAG(BHQ-1) |  |
| **5** | HAdV | Hexon protein | J01917 | Fwd: GCCACGGTGGGGTTTCTAAACTT | [36] |
|  |  | (18858-18989) |  | Rev: GCCCCAGTGGTCTTACATGCACATC |  |
|  |  |  |  | Pr: (6-FAM)-TGCACCAGACCCGGGCTCAGGTACTCCGA-(TAMRA) |  |
|  | HBoV | NonStructural protein | DQ000495 | Fwd: GCACAGCCACGTGACGAA | [37] |
|  |  | (2391-2466) |  | Rev: TGGACTCCCTTTTCTTTTGTAGGA |  |
|  |  |  |  | Pr: (JOE)-TGAGCTCAGGGAATATGAAAGACAAGCATCG-(BHQ-1) |  |

^a^ Fwd=Forward; Rev=Reverse; Pr=Probe; 6-FAM=6-Carboxyfluorescein; TAMRA=Tetramethylrhodamine; JOE=6-carboxy-dichloro-dimethoxyfluorescein; BHQ-1, -2=Black Hole Quencher™-1, -2; ROX=6–Carboxyl-X-Rhodamine; Cy5=Cyanin-5.
