# Supplementary material for: Viral Etiology of Influenza-Like Illnesses in Antananarivo, Madagascar, July 2008 to June 2009
Source: PLoS One. 2011 Mar 3;6(3):e17579. doi: 10.1371/journal.pone.0017579 (PMC3048401; doi:10.1371/journal.pone.0017579)
Supplement: Table S2 — Distribution of age groups related to each type of major co-infections. (DOC) [file pone.0017579.s003.doc]

**Table S3**: Distribution of age groups related to each type of major co-infection pairs.

| **Age groups (years)** | **Viruses (%)** | | | | |
| --- | --- | --- | --- | --- | --- |
|  | **HRV/RSV** | **FLUAV/HRV** | **FLUAV/RSV** | **FLUAV/HCoV-OC43** | **Others^a^** |
|  | **N=15** | **N=14** | **N=8** | **N=5** | **N=27** |
| 0-4 | 13 (86.7) | 6 (43.0) | 5 (62.5) | 1 (20.0) | 20 (74.1) |
| 5-9 | 2 (13.3) | 1 (7.1) | 3 (37.5) | 0 (0.0) | 1 (3.7) |
| 10-14 | 0 (0.0) | 3 (21.4) | 0 (0.0) | 1 (20.0) | 0 (0.0) |
| 15-19 | 0 (0.0) | 1 (7.1) | 0 (0.0) | 1 (20.0) | 2 (7.4) |
| >= 20 | 0 (0.0) | 3 (21.4) | 0 (0.0) | 2 (40.0) | 4 (14.8) |

N=total number of cases

^a^ for other co-infection pairs, number of cases were equal or less than 2
